# Variation in area proportion and mechanical properties between different subregions of ACL insertion: An in vitro biomechanical study in a porcine model

**DOI:** 10.1002/jeo2.70470

**Published:** 2025-10-31

**Authors:** Kaixin He, Qingqing Yang, Qinyi Shi, Huizhi Wang, Cheng‐Kung Cheng

**Affiliations:** ^1^ School of Biomedical Engineering Shanghai Jiao Tong University Shanghai China; ^2^ Center for Intelligent Medical Equipment and Devices, School of Biomedical Engineering, Division of Life Sciences and Medicine University of Science and Technology of China Hefei China; ^3^ Suzhou Institute for Advanced Research University of Science and Technology of China Suzhou China

**Keywords:** anterior cruciate ligament insertion, area proportion, functional region, mechanical property, stress level, subregion

## Abstract

**Purpose:**

To quantitatively evaluate differences in area proportions and Young's modulus among anatomical subregions of the anterior cruciate ligament (ACL) insertion, including direct and indirect insertions as well as anteromedial (AM) and posterolateral (PL) bundles, and to assess their impact on local stress distribution.

**Methods:**

Micro‐CT was utilized to quantify the area proportions of four anatomically defined subregions of porcine ACL insertions (*n* = 6). Mechanical testing was conducted to assess the Young's modulus of each region (*n* = 6). Finite element analysis was performed to evaluate how variations in regional area proportions (30%, 50% and 70% for the direct insertion) and mechanical properties (homogeneous vs. heterogeneous distributions) influence load distribution at the insertion.

**Results:**

The direct region occupied a significantly larger area proportion than the indirect region (54% vs. 46%, *p* < 0.01), and the AM bundle covered a significantly greater area than the PL bundle (65% vs. 35%, *p* < 0.01). The indirect region demonstrated a significantly higher Young's modulus than the direct region (12.0 vs. 6.8 MPa, *p* < 0.01), while no significant difference was observed between AM and PL bundles (8.4 vs. 10.4 MPa). Finite element results indicated that stress distribution at the insertion became more uniform when the direct and indirect regions had comparable area proportions, and incorporating regional heterogeneity in mechanical properties resulted in increased force transmission through the indirect region.

**Conclusions:**

Distinct regional differences in area proportions and Young's moduli were found at the ACL insertion, and these characteristics substantially affect local stress distribution.

**Level of Evidence:**

Level N/A.

## Graphical abstract

This study quantitatively evaluates the area proportions and mechanical properties of anatomical subregions within the porcine anterior cruciate ligament (ACL) insertion. Significant regional differences in area and Young's modulus were observed, and finite element analysis revealed their critical roles in local stress distribution. These findings provide insights for improving anatomical reconstruction of the ACL.

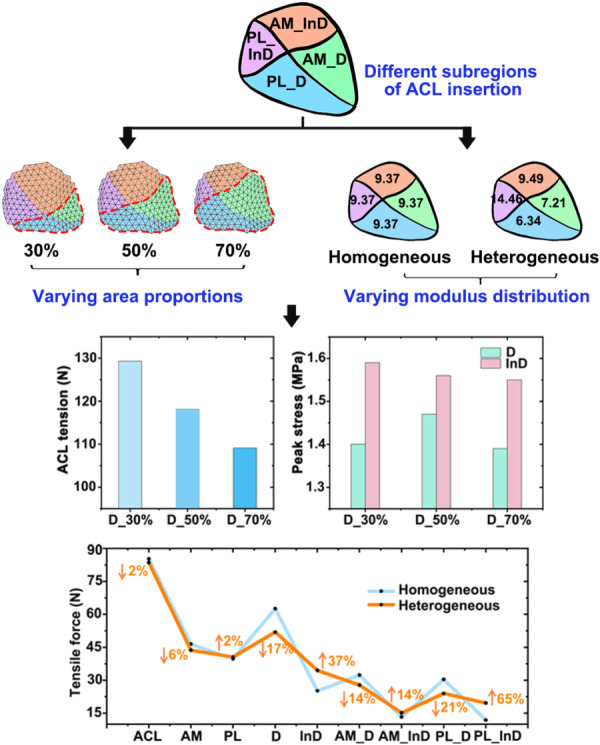

**Abbreviations**
ACL: anterior cruciate ligament
ACLR: ACL reconstruction
AM: anteromedial
D: direct
InD: indirect
LCL: lateral collateral ligament
MCL: medial collateral ligament
PCL: posterior cruciate ligament
PL: posterolateral

## INTRODUCTION

The anterior cruciate ligament (ACL) is a key stabilizer of the knee joint [17], but is highly prone to injury [6]. Although ACL reconstruction (ACLR) is the standard treatment, complications related to the graft‐bone interface remain common, such as tunnel enlargement and graft rupture [16]. These issues may be partly due to inadequate replication of the native ligament insertion in anatomical structure, material properties, and mechanical function. Unlike homogeneous tendon grafts, the native ACL insertion shows clear regional differentiation, including anteromedial (AM) and posterolateral (PL) bundles [4] and direct and indirect insertions [14]. Understanding how these functional regions differ in area proportion, Young's modulus and mechanical roles is essential for improving reconstruction strategies and informing biomimetic graft design.

While previous studies have quantified the areas of these subregions at both femoral [7, 9, 12] and tibial [13] insertion sites, they typically reported averaged absolute values across subjects, which may overlook the subject‐specific variation in area proportions. Our previous anatomical dissections revealed marked inter‐individual differences in regional area proportions, underscoring the need to explore their biomechanical implications [17].

Previous studies found no significant difference in Young's modulus between porcine AM and PL bundles (111 ± 30 vs. 123 ± 46 MPa) [19], but differences between direct and indirect insertions remain poorly understood. Most computational models assume a mechanically homogeneous ACL [8], overlooking regional variations. However, empirical observations by the present authors suggest that the indirect insertion appears stiffer than the direct insertion, warranting further investigation into how this heterogeneity influences mechanical behaviour at the insertion.

The AM bundle resists anterior–posterior tibial translation functionally, whereas the PL bundle contributes to rotational stability [14]. Kawaguchi et al. [3] reported that the direct insertion bears 66%–84% of joint loading. In contrast, Sabzevari et al. [9] demonstrated that the presence of the indirect insertion increased the failure load of the knee joint from 392 to 3599 N, significantly enhancing resistance to anterior tibial translation. The variability in regional area proportions and Young's moduli may also contribute to inconsistencies observed in the above biomechanical findings.

The objectives of this study were: (1) to quantify the area proportions and Young's modulus of the AM and PL bundles as well as the direct and indirect regions of the ACL insertion; and (2) to evaluate how regional variations in area proportion and Young's modulus influence stress distribution. Given the anatomical and functional similarity between porcine and human ACLs [10], and that the porcine knee joints are more accessible, cadaveric porcine specimens were used in this study. This study may advance our understanding of region‐specific structure‐material‐function relationships at the ligament insertion and provide a foundation for optimizing ACL reconstruction strategies and designing biomimetic ligament grafts.

## METHODS

**Figure 1:**
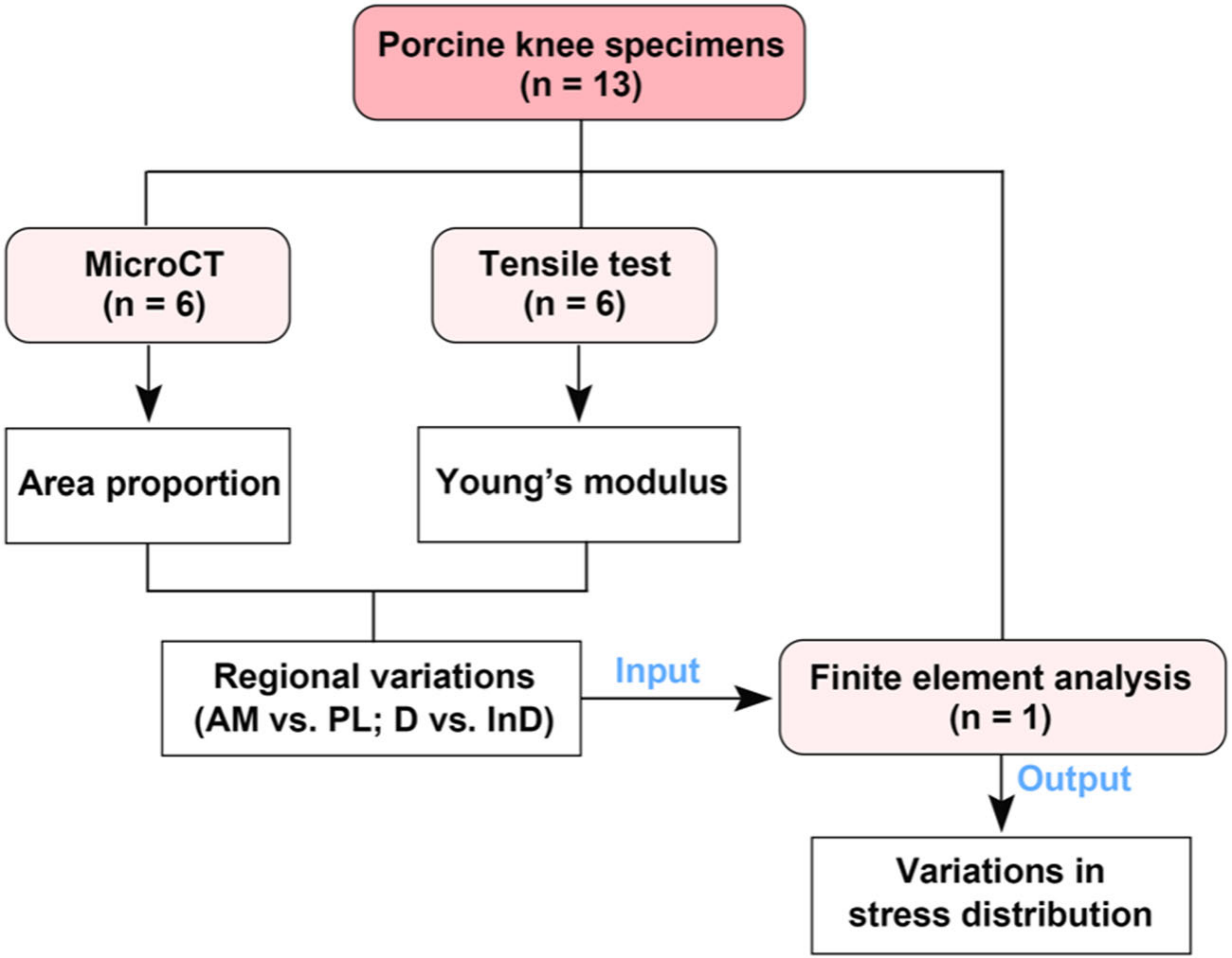
Flowchart of the study design. AM, anteromedial bundle; D, direct insertion; InD, indirect insertion; PL, posterolateral bundle; *n*, number of samples included in each experimental section.

The overall study design is summarized in the flowchart shown in Figure 1.

### Specimen acquisition and preparation

**Figure 2:**
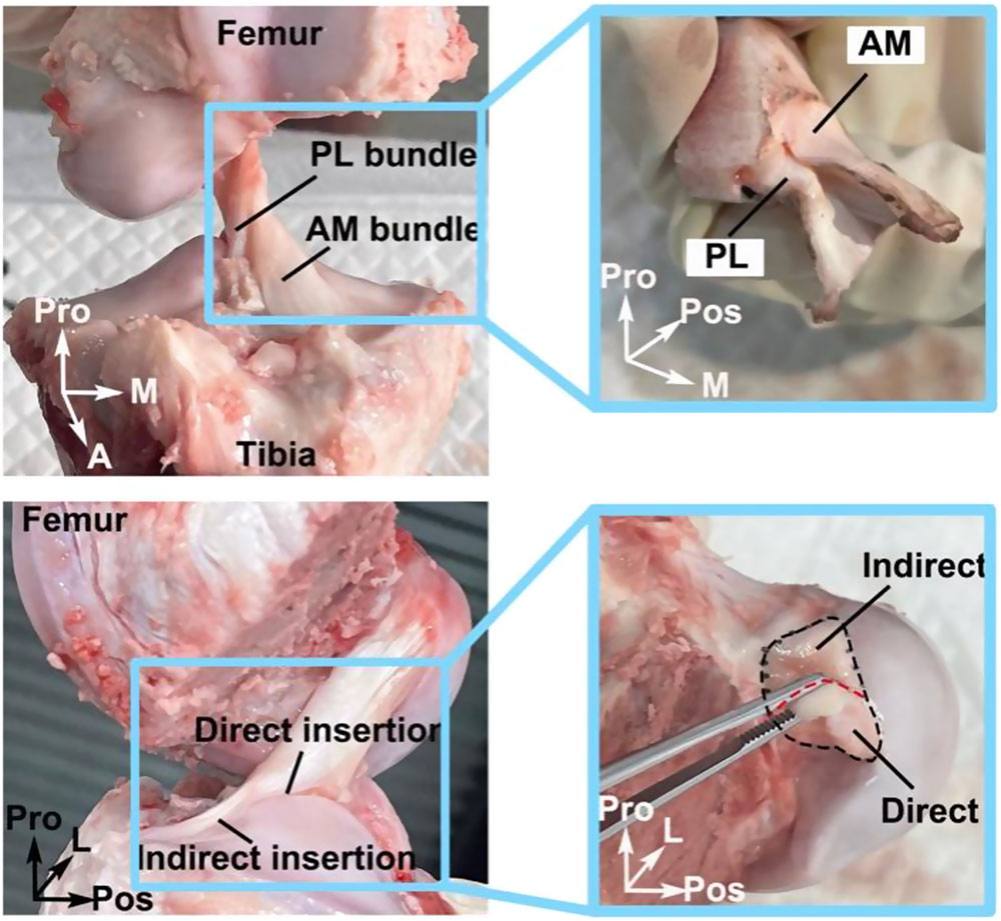
Prepared porcine knee specimen showing the anteromedial (AM) and posterolateral (PL) bundles, as well as the direct and indirect regions of the ACL insertion. A, anterior; ACL, anterior cruciate ligament; L, lateral; M, medial; Pos, posterior; Pro, proximal.

The project was approved by the Institutional Animal Care and Use Committee of Shanghai Jiao Tong University (Approval Code: 202101260). Right knee joints from 13 pigs (Duroc‐Albion‐York cross, male, 1 year old) were obtained from a licensed slaughterhouse and stored at −20°C within 24 h postmortem. One specimen was preserved intact for the development of magnetic resonance imaging (MRI) and a finite element model. The other samples were thawed at room temperature for 12 h before dissection. The patella and surrounding soft tissues were removed to expose the intra‐articular structures. The medial femoral condyle was excised after careful removal of the posterior cruciate ligament and menisci. Only specimens with visually intact and healthy ACLs were included for subsequent analysis. Each final specimen consisted of the femur, ACL (including the AM and PL bundles), and tibia, with both direct and indirect insertions preserved (Figure 2).

### Quantification of regional area proportions

**Figure 3:**
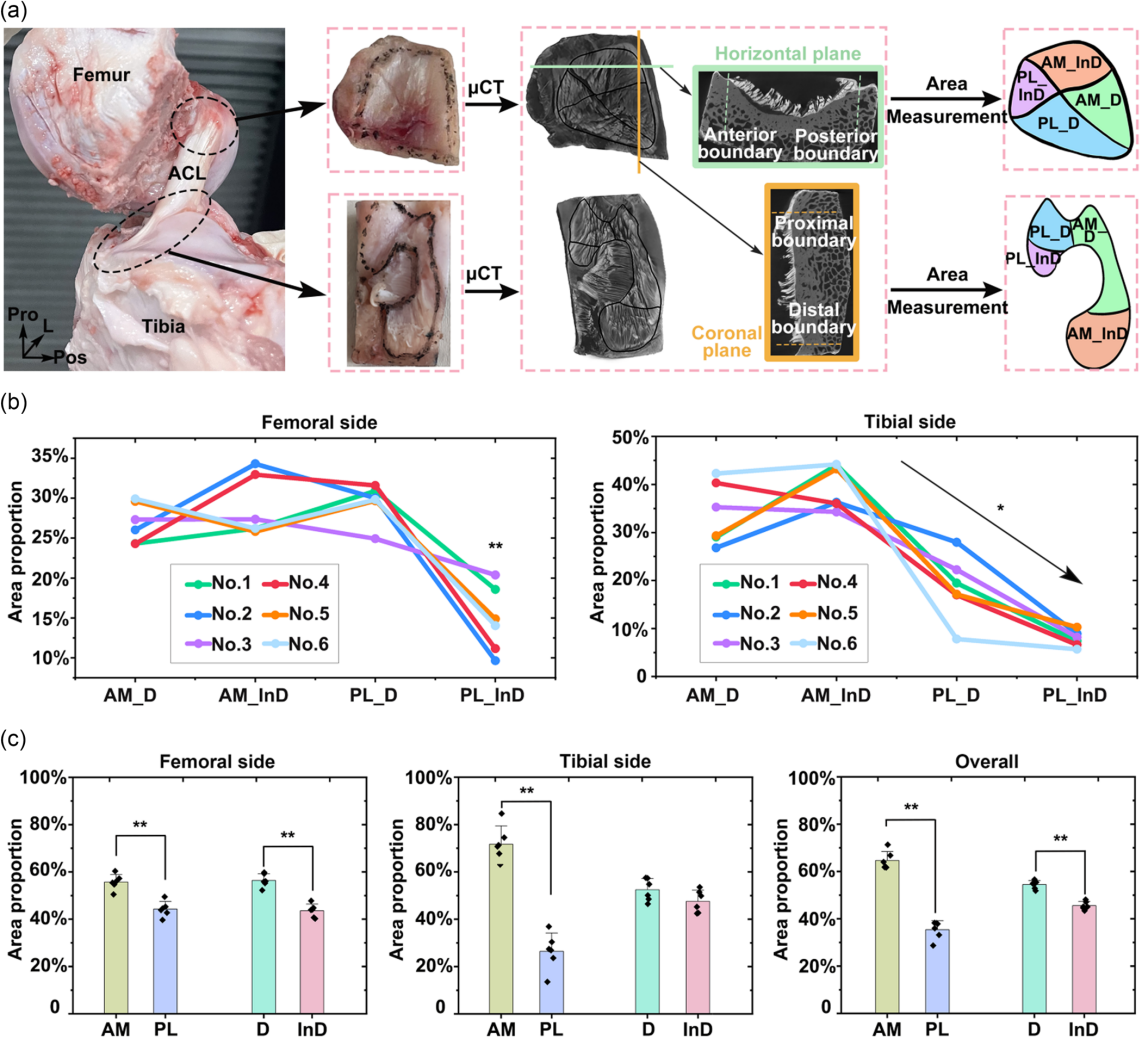
Quantification of regional area proportions. (a) Schematic illustration of sample preparation and regional area measurement using micro‐CT analysis. (b) Quantified area proportions of anatomically distinct subregions. (c) Statistical comparisons of area proportions between the anteromedial (AM) and posterolateral (PL) bundles, and between the direct (D) and indirect (InD) insertions. ACL, anterior cruciate ligament; AM_D, direct insertion of the AM bundle; AM_InD, indirect insertion of the AM bundle; L, lateral; PL_D, direct insertion of the PL bundle; PL_InD, indirect insertion of the PL bundle; Pos, posterior; Pro, proximal. *Significant difference with *p* < 0.05; **Significant difference with *p* < 0.01.

Six samples were used to quantify the proportions of the regional area. The samples were initially fixed in 2.5% glutaraldehyde for 2 h to preserve the native orientation of ligament fibres. The fibres were then carefully excised from the bone using a scalpel, leaving a 1–2 mm remnant attached to facilitate complete dehydration of the residual fibres. The contours of the ligament insertion were manually marked on both the femoral and tibial sides (Figure 3a), and an electric reciprocating saw was used to cut the bone along the marked outlines. After trimming, the samples were fixed again in 2.5% glutaraldehyde at room temperature for 6 h, followed by phosphate‐buffered saline rinsing for 30 min. Dehydration was performed using a graded ethanol series (20%, 40%, 60%, 80%, and 100%, each for 30 min). Samples were stained sequentially for two nights in 70% ethanol + 1% phosphotungstic acid and 80% ethanol + 1% PTA solutions, followed by further dehydration in 90%, 95%, and 100% ethanol solutions. Critical point drying was then performed and the dried samples were scanned using micro‐CT (Zeiss Xradia 520 Versa; Carl Zeiss AG) at a resolution of 25 μm, with ×0.4 optical magnification, and an exposure time of 1.2 s at 60 kV tube voltage and 5 W power. Three‐dimensional reconstructions were performed using Dragonfly software (ORS).

The contours of the ligament insertion were delineated in the reconstructed 3D models. For the femoral insertion, anterior and posterior boundaries were identified on the horizontal plane. In contrast, proximal and distal boundaries were identified on the coronal plane (Figure 3a). The complete femoral insertion was outlined on the sagittal plane. For the tibial insertion, anterior and posterior boundaries were identified on the sagittal plane, and medial and lateral boundaries were identified on the coronal plane. The entire tibial insertion was outlined on the horizontal plane. Based on the trajectories of prominent synovial membranes (Figure 3a), the insertions were divided into four subregions: AM_D (direct insertion of the AM bundle), AM_InD (indirect insertion of the AM bundle), PL_D (direct insertion of the PL bundle) and PL_InD (indirect insertion of the PL bundle). The area of each subregion was quantified on the corresponding insertional plane using Dragonfly, as previously described [18], and regional area proportions were subsequently calculated. All measurements were performed by an author experienced with the entire procedure. The reliability of anatomical area measurements on micro‐CT images has been validated, demonstrating excellent intra‐ and inter‐observer agreement, with an intraclass correlation coefficient exceeding 0.99 [18].

### Quantification of regional tensile Young's modulus

**Figure 4:**
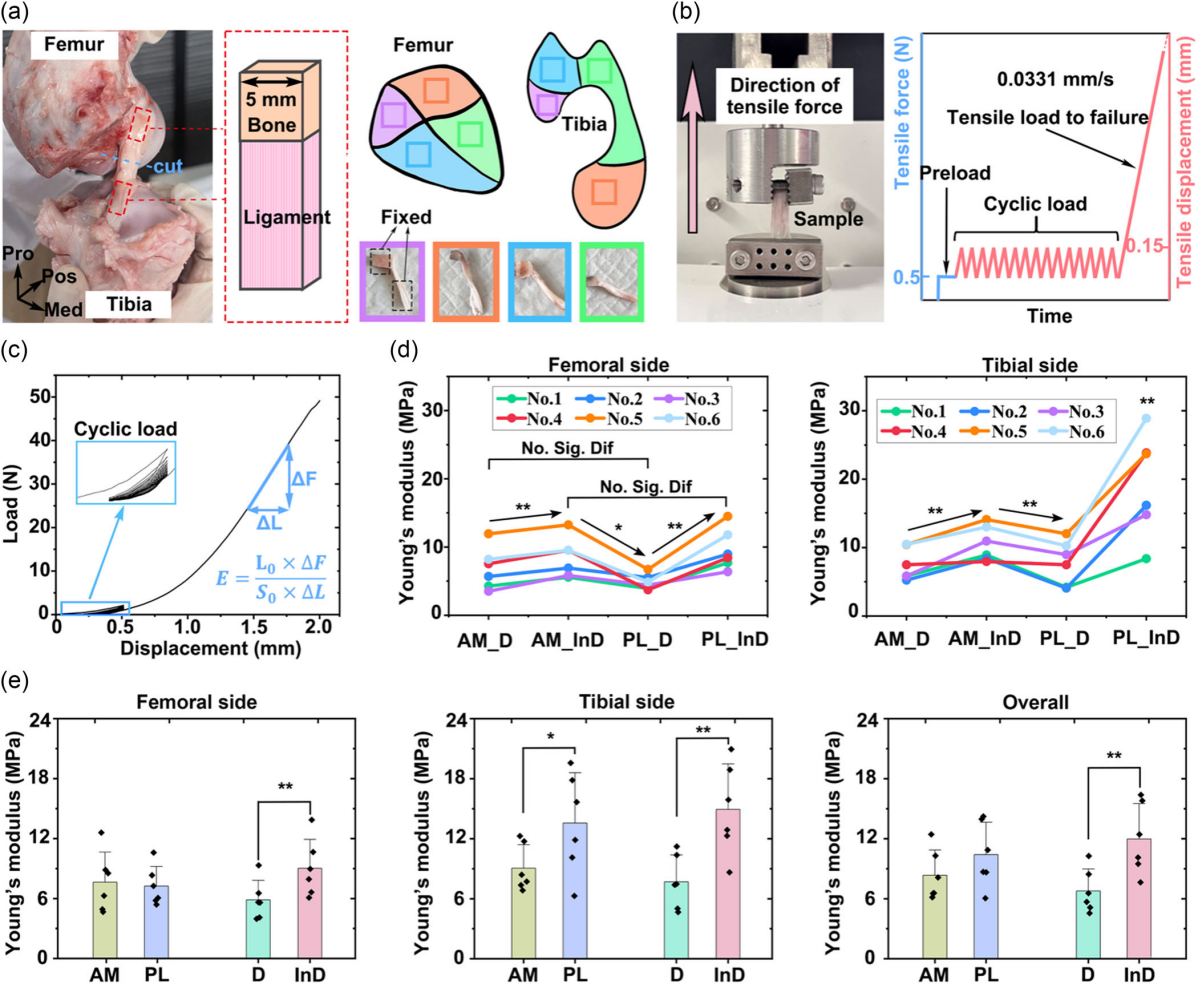
Quantification of regional tensile Young's modulus. (a) Schematic illustration of sample preparation. The purple, orange, blue and green boxes represent the indirect insertion of the PL bundle, the indirect insertion of the AM bundle, the direct insertion of the PL bundle, and the direct insertion of the AM bundle, respectively. (b) Mechanical testing setup for quantifying regional tensile Young's modulus. (c) A representative displacement‐load curve was obtained for Young's modulus calculation. (d) Quantified tensile Young's modulus of distinct subregions. (e) Statistical comparisons of tensile Young's modulus between the anteromedial (AM) and posterolateral (PL) bundles, and between the direct (D) and indirect (InD) insertions. AM_D, direct insertion of the AM bundle; AM_InD: indirect insertion of the AM bundle; Med, medial; No. X, sample ID; Pos, posterior; Pro, proximal; PL_D, direct insertion of the PL bundle; PL_InD, indirect insertion of the PL bundle. *Significant difference with *p* < 0.05; **Significant difference with *p* < 0.01.

Six samples were used to quantify the regional tensile Young's modulus. The ACL was transversely sectioned at its mid‐substance. The ligament insertions were further divided into four anatomically distinct subregions mentioned above (AM_D, AM_InD, PL_D and PL_InD) (Figure 4a). To obtain standardized specimens, each sample was prepared as a square cross‐sectioned cuboid containing approximately 10 mm ligament and 5 mm bone. The central area of each subregion was sampled with a side length of 5 mm to ensure consistency, as illustrated by the colour‐coded boxes in Figure 4a.

Uniaxial tensile testing was conducted using a mechanical testing machine (Orthotek Lab). The ends of the bone and ligament were fixed by the upper and lower clamps, respectively, with the sample aligned along its anatomical fibre orientation. Both ends were clamped over a length of 5 mm, leaving an unsupported ligament section of 5 mm in the middle (Figure 4a). A preload of 0.5 N was applied, followed by 15 cycles of loading‐unloading between 0 and 0.15 mm at a speed of 0.0331 mm/s [5]. After preconditioning, the specimen was stretched at the same rate until the loading reached the maximum measurable tensile force of the testing device (50 N), at which point the loading was terminated due to the sensor's capacity limit (Figure 4b). The tensile Young's modulus was calculated from the linear portion of the resulting displacement‐load curve using the measured cross‐sectional area ($S_{0}$) and initial length ($L_{0}$) of each sample (Figure 4c).

### Development and validation of a finite element model of the porcine knee

**Figure 5:**
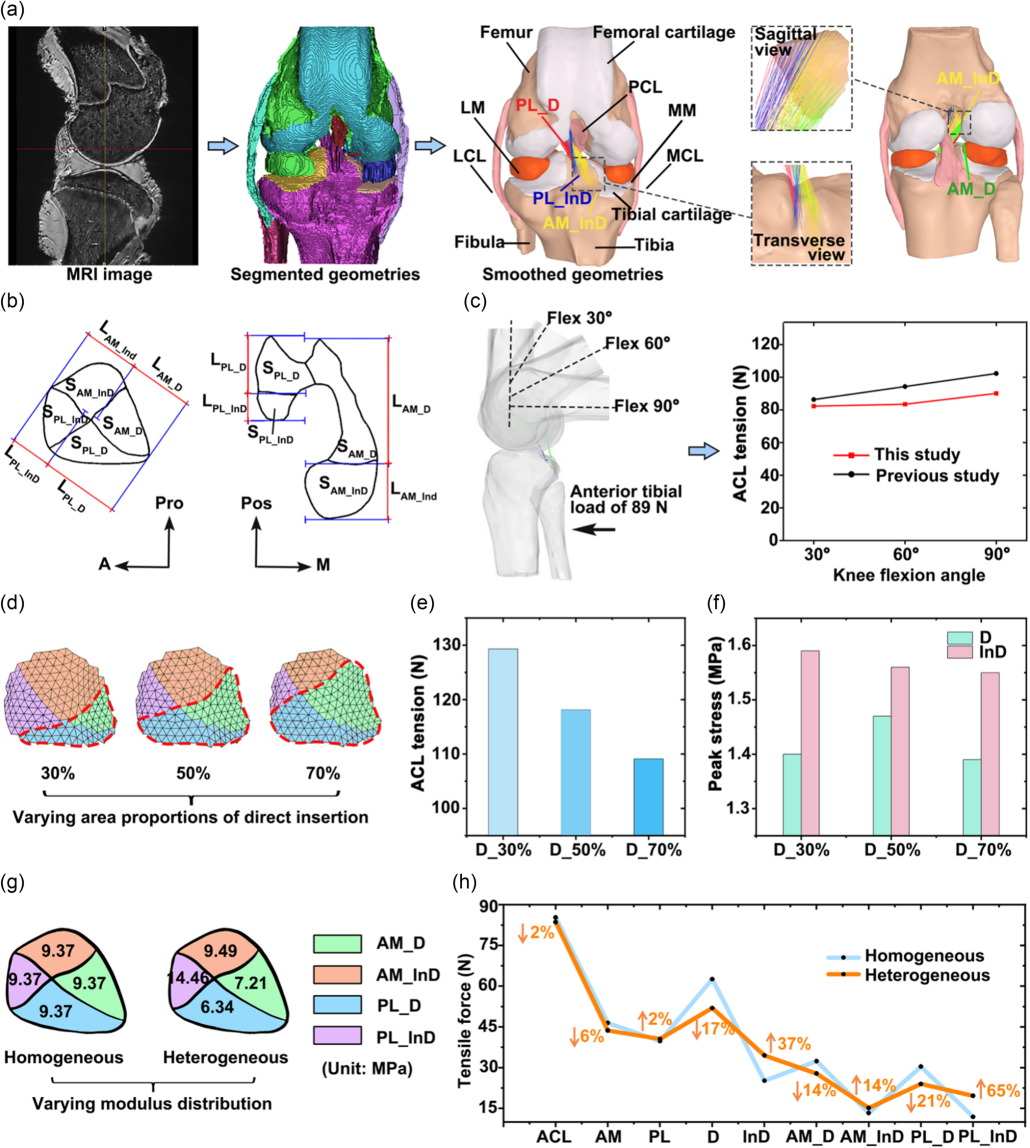
Finite element analysis investigating the influence of regional area proportions and tensile Young's modulus on load distribution at the ACL insertion. (a) Schematic of model development. (b) Measure area and length ratios of direct and indirect insertions of the AM and PL bundles for anatomical model validation. (c) Boundary and loading conditions and model‐predicted ACL tension for biomechanical model validation. (d) Definition of varying area proportions of the direct insertion in the model. (e) Change in ACL tension with varying area proportions of the direct insertion. (f) Change in peak stress within the direct and indirect insertions as the area proportions of the direct insertion vary. (g) Homogeneous and heterogeneous distribution of regional tensile Young's modulus as defined in the model. (h) Differences in regional tensile forces between homogeneous and heterogeneous material models. A, anterior; ACL, anterior cruciate ligament; AM_D, direct insertion of the AM bundle; AM_InD, indirect insertion of the AM bundle; LCL, lateral collateral ligament; LM, lateral meniscus; M, medial; MCL, medial collateral ligament; MM, medial meniscus; MRI, magnetic resonance imaging; PL_D, direct insertion of the PL bundle; PL_InD, indirect insertion of the PL bundle; Pos, posterior; Pro, proximal.

A porcine knee joint was fixed in 10% neutral‐buffered formalin at full extension for 2 weeks and subsequently scanned using MRI (TE = 4.3 ms, TR = 380 ms, resolution = 0.4 × 0.4 mm, slice thickness = 0.4 mm) (Figure 5a). The MRI images were segmented using Mimics 21.0 (Materialise N. V.) to construct a finite element model of the joint. The model included the femur, tibia, fibula, ACL, posterior cruciate ligament (PCL), medial collateral ligament (MCL), lateral collateral ligament (LCL), menisci, and femoral and tibial cartilages. The geometry was meshed using HyperMesh 2019 (Altair Engineering). The ACL was modelled using truss units and was divided into four bundles (AM_D, AM_InD, PL_D, PL_InD), with Young's modulus assigned according to results from mechanical testing. Bone (Young's modulus of 389 MPa, Poisson's ratio of 0.33), cartilage (Young's modulus of 5 MPa, Poisson's ratio of 0.46), PCL, MCL and LCL (Young's modulus of 10 MPa, Poisson's ratio of 0.4) were defined as isotropic linear elastic materials [15]. The meniscus (*E*_θ_ = 125 MPa, *E*_R_ = *E*_Z_ = 27.5 MPa, *G*_θR_ = G_θZ_ = 2 MPa, *G*_RZ_ = 10.34, *V*_θR_ = *V*_θZ_ = 0.1, *V*_RZ_ = 0.33) was defined as orthotropic linearly elastic. A frictionless sliding contact was assigned between the femoral and tibial cartilage and between the femoral cartilage and meniscus, allowing the two contact surfaces to slide without penetrating each other. Tie constraints were applied between the two ends of each ligament and their corresponding bone insertion to ensure displacement continuity at the insertion [15]. The cartilage and corresponding bone surface were also tied. All components were meshed using first‐order tetrahedral elements. Abaqus/CAE 6.14‐2 (Simulia, Inc.) was used for subsequent finite element analysis. The entire modelling procedure was performed by one of the authors with expertise in musculoskeletal modelling and finite element analysis.

A mesh convergence test was conducted to optimize the element size, ensuring it was sufficiently small for accurate computation while avoiding an excessively high element count that would lead to unnecessary computational cost. In this test, a 2 mm anterior translational load was applied to the tibia while the femur was fixed at full extension to calculate the in situ force of the ACL. The element size was reduced iteratively until the change in calculated ACL force became negligible. The final element size was 1 mm, resulting in 574,085 elements.

The model measured the anatomical dimensions of the ACL insertion, including the subregional area and length ratios (Figure 5b), and compared them with those obtained from micro‐CT to evaluate the anatomical accuracy of the multi‐bundle ACL representation.

To validate the model's biomechanical accuracy, an anterior tibial load of 89 N was applied at knee flexion angles of 30°, 60° and 90° (Figure 5c). The resulting ACL tensions were compared with experimental data reported in the literature [2].

### Influence of regional area proportions and tensile Young's modulus on load distribution across functional regions of the ACL insertion

Three models were developed to simulate the ACL insertion with varying area proportions between the direct and indirect regions. Based on microCT measurements, the direct insertion was modelled to occupy 30%, 50% and 70% of the total area, respectively (Figure 5d). An anterior drawer test was simulated by applying a 5 mm anterior tibial translation at 30° of knee flexion. The resulting ACL tensile force and peak stress within both the direct and indirect insertions were recorded and compared across the three models to evaluate the effect of regional area proportions on load distribution at the ACL insertion.

To further assess the influence of regional mechanical properties, two additional models were constructed with homogeneous and heterogeneous distributions of Young's modulus across the functional regions of the ACL insertion (Figure 5g). The homogeneous model applied a uniform modulus equal to the average of all four subregions (AM_D, AM_InD, PL_D, PL_InD) throughout the insertion. The region‐specific modulus derived from uniaxial mechanical testing was assigned to each corresponding subregion in the heterogeneous model. An anterior tibial translation of 5 mm at 30° of flexion was again used to simulate the anterior drawer test. The resulting tensile forces in the entire ACL, as well as in the AM, PL, direct, indirect, and all four subregions (AM_D, AM_InD, PL_D, PL_InD), were calculated and compared between the two models to investigate the impact of material heterogeneity on load distribution across the ACL insertion.

### Statistical analysis

Before the formal experiments, pre‐experiments were conducted using three samples to estimate the required sample sizes for measuring regional area proportions and tensile Young's modulus. Power analysis was performed using PASS 15 software (NCSS, LLC). A minimum sample size of four was required to detect a statistically significant difference in area proportions between the AM and PL bundles with a significance level (*α*) of 0.05 and statistical power of 0.90. The required sample sizes for detecting significant differences between the direct and indirect insertions in area proportion and Young's modulus were six and four, respectively.

For each bundle (AM and PL), the total value was calculated as the sum of its direct and indirect insertion components. Similarly, the total values for the direct and indirect insertion regions were calculated by summing the corresponding values from both the AM and PL bundles. Values from the femoral and tibial sides were averaged to compare overall behaviour.

All statistical analyses were conducted in IBM SPSS Statistics 25 (IBM Corp.). Paired *t*‐tests assessed the significance of differences between the AM and PL bundles and the direct and indirect insertion regions. The Shapiro–Wilk normality test indicated that the differences between all paired groups subjected to the paired *t*‐test followed a normal distribution. A post hoc power analysis verified the adequacy of statistical power supporting the main conclusions.

## RESULTS

### Quantification of regional area proportions

The area proportions of the AM_D, AM_InD, PL_D, and PL_InD subregions for both the femoral and tibial sides are shown in Table 1. At the femoral side, the PL_InD region exhibited a significantly smaller area proportion than all other subregions (*p* < 0.01) (Figure 3b). At the tibial side, no significant difference was found between the AM_D and AM_InD regions (*p* = 0.134). In contrast, the PL_D region was significantly smaller than both AM bundle subregions (*p* < 0.05), and the PL_InD region was substantially smaller than the PL_D region (*p* < 0.01).

**Table 1:** Measured area proportions across subregions.

	AM_D	AM_InD	PL_D	PL_InD
Femoral	27% ± 2%	29% ± 3%	30% ± 2%	15% ± 4%
Tibial	34% ± 6%	40% ± 4%	19% ± 6%	8% ± 1%

Abbreviations: AM_D, direct insertion of the AM bundle; AM_InD, indirect insertion of the AM bundle; PL_D, direct insertion of the PL bundle; PL_InD, indirect insertion of the PL bundle.

When grouped by functional bundles, the AM bundle exhibited a significantly larger area proportion than the PL bundle on both the femoral and tibial sides, as well as in the combined analysis (*p* < 0.01, power = 1 for the combined analysis, Figure 3c). The direct insertion accounted for a significantly greater proportion than the indirect insertion at the femoral side and in the overall measurement (*p* < 0.01, power = 1 for the overall measurement). No significant difference was detected between the direct and indirect insertions at the tibial side.

### Quantification of regional tensile Young's modulus

The tensile Young's modulus of the AM_D, AM_InD, PL_D, and PL_InD subregions for both the femoral and tibial sides are presented in Table 2. No significant differences were observed between AM_D and PL_D regions (*p* = 0.1), or between AM_InD and PL_InD regions (*p* = 0.08) (Figure 4d). However, the AM_InD region exhibited significantly higher modulus than the AM_D region (*p* < 0.01), and the PL_D region exhibited substantially lower value than both AM_InD and PL_InD regions (*p* < 0.05). At the tibial side, no significant difference was found between AM_D and PL_D regions (*p* = 0.72). The PL_InD region exhibited significantly higher modulus than all other subregions (*p* < 0.05), and the AM_InD region exhibited significantly higher modulus than both AM_D and PL_D regions (*p* < 0.01).

**Table 2:** Measured Young's modulus in MPa across subregions.

	AM_D	AM_InD	PL_D	PL_InD
Femoral	6.9 ± 2.8	8.4 ± 2.7	4.9 ± 1.0	9.6 ± 2.7
Tibial	7.6 ± 2.2	10.6 ± 2.3	7.8 ± 3.0	19.3 ± 6.9

Abbreviations: AM_D, direct insertion of the AM bundle; AM_InD, indirect insertion of the AM bundle; PL_D, direct insertion of the PL bundle; PL_InD, indirect insertion of the PL bundle.

When grouped by functional bundles, the AM bundle exhibited significantly lower modulus than the PL bundle only at the tibial side (*p* < 0.05, power = 0.81), with no significant difference observed at the femoral side or in the overall comparison (*p* = 0.55 and 0.073, Figure 4e). The indirect insertion exhibited significantly greater modulus than the direct insertion at both femoral and tibial sides, as well as in the overall analysis (*p* < 0.01, power = 1 for the overall analysis).

### Anatomical and biomechanical validation of the finite element model

The area and length ratios of the direct and indirect insertions of the AM and PL bundles measured in the finite element model were all within the range of cadaveric experimental values (Table 3), supporting the anatomical fidelity of the multi‐bundle ACL representation.

**Table 3:** Area and length ratios of the direct and indirect insertions of AM and PL bundles measured from the knee model and cadaveric experiments.

			AM_D/AM	AM_InD/AM	PL_D/PL	PL_InD/PL
Area ratio	Femoral	Experiment	41%–51%	49%–59%	56%–64%	56%–44%
		Model	44%	56%	60%	40%
	Tibial	Experiment	42%–56%	44%–57%	61%–76%	24%–38%
		Model	49%	51%	68%	32%
Length ratio	Femoral	Experiment	51%–61%	39%–49%	65%–75%	25%–35%
		Model	53%	47%	71%	29%
	Tibial	Experiment	59%–71%	29%–41%	61%–75%	25%–39%
		Model	63%	37%	65%	35%

Abbreviations: AM, anteromedial; AM_D, direct insertion of the AM bundle; AM_InD, indirect insertion of the AM bundle; PL, posterolateral; PL_D, direct insertion of the PL bundle; PL_InD, indirect insertion of the PL bundle.

Under anterior tibial loading, ACL tension increased progressively with knee flexion angle, consistent with trends reported in the previous study [2] (Figure 5c). The difference in resultant ACL tension between the model and previously reported experimental data ranged from 5% to 12%, which may be attributed to inter‐individual differences.

### Influence of regional area proportions on load distribution at the ACL insertion

As the area proportion of the direct insertion increased from 30% to 70%, the overall ACL tension decreased from 129 to 109 N (Figure 5e). The corresponding peak von Mises stresses within the direct and indirect insertions are presented in Figure 5f. Across all conditions, the indirect insertion consistently exhibited higher peak stress than the direct insertion. Notably, the difference in peak stress between the two regions was minimized when the direct insertion accounted for 50% of the total insertion, indicating a more balanced load bearing under this configuration.

### Influence of regional tensile Young's modulus on load distribution at the ACL insertion

The force distribution across the ACL insertion differed markedly between models with homogeneous and heterogeneous material properties. Compared to the homogeneous model, the model with individually assigned Young's modulus for each subregion produced a 2% reduction in total ACL tension. Notably, the heterogeneous model led to a 37% increase in force distributed to the indirect insertion and a 17% decrease at the direct insertion. Subregion‐level analysis revealed a 14% and 21% reduction in tensile force at the AM_D and PL_D regions, respectively, while forces increased by 14% and 65% in the AM_InD and PL_InD regions. In contrast, force distribution between the AM and PL bundles remained relatively stable, with variations of only 2%–6%.

## DISCUSSION

This study revealed that the area proportion of the direct insertion was significantly greater than that of the indirect insertion, and the AM bundle occupied a substantially larger area than the PL bundle. Regarding mechanical properties, the Young's modulus of the indirect insertion was significantly higher than that of the direct insertion. At the same time, no significant difference was observed between the AM and PL bundles. Finite element analyses demonstrated that incorporating region‐specific material heterogeneity markedly increased tensile force at the indirect insertion. Furthermore, the disparity in peak stress between the direct and indirect insertions was minimized when their area proportions were approximately equal. Although a porcine model was used in this study, the findings underscore the importance of accurately replicating region‐specific material properties and area distributions in ACL reconstruction to restore appropriate mechanical function of the ligament insertion.

Although no prior study has systematically compared area proportions across regions, the current finding that the AM bundle has a larger area than the PL bundle is consistent with observations by Siebold et al. [11]. Similarly, previous observations of a smaller indirect insertion area relative to the direct insertion at the femoral ACL insertion [7] align with our findings. Regarding mechanical properties, our earlier qualitative observations suggested that the indirect insertion was firmer to palpation than the direct insertion, a finding now quantitatively confirmed by the significantly higher Young's modulus of the indirect region. In addition, our finding of no significant difference in Young's modulus between the AM and PL bundles is consistent with a previous biomechanical study [19].

Interestingly, the PL_InD region exhibited the smallest area proportion but the highest Young's modulus among the four subregions on both the femoral and tibial sides. This may suggest that, to maintain sufficient mechanical performance despite its small area, this subregion undergoes a disproportionately greater increase in material stiffness. A similar inverse relationship between area proportion and Young's modulus was observed at the broader regional level, where the indirect insertion showed a smaller area but greater modulus than the direct insertion. A comparable (though nonsignificant) trend was also noted between the AM and PL bundles.

The overall tensile force of the ACL decreased as the area proportion of the direct insertion increased (Figure 5e), likely due to the lower modulus of the direct insertion, which compromised its load‐bearing capacity. We speculate that during growth or adaptive responses to mechanical loading, the indirect insertion proportion may increase to enhance load‐bearing ability. This hypothesis is supported by previous findings [7] showing that older individuals have a larger indirect insertion area and a smaller direct insertion area than younger individuals. Moreover, our results indicated that peak stress in the indirect insertion consistently exceeded that in the direct insertion, and the stress disparity widened with increasing differences in area proportions. These observations suggest that shifts in the boundary between direct and indirect regions influence the magnitude and distribution of regional loads. When the boundary is positioned near the midpoint, the disparity in peak stress is minimized, suggesting a lower risk of regional mechanical failure. This interpretation is consistent with previous studies [1] reporting no significant difference in area proportions between direct and indirect insertions in human cadaver knees with a median age of 82.5 years.

Compared to the homogeneous material model, assigning region‐specific Young's modulus led to a redistribution of load, significantly reducing the bearing force at the direct insertion while increasing that at the indirect insertion. This finding suggests that the indirect insertion may have biomechanically adapted to withstand higher loads, thereby protecting the structurally weaker direct insertion from mechanical failure. These findings emphasize the importance of incorporating the functional role of the indirect insertion into the restoration of knee joint biomechanics and the design of ACL reconstruction strategies.

Overall, these findings emphasize that individual variations in area proportions and material properties across distinct functional regions of the ACL insertion critically influence its biomechanical behaviour and mechanical failure risk. Therefore, careful evaluation and restoration of these anatomical and mechanical features are essential to maintain the functional integrity of the soft‐hard tissue interface during ligament reconstruction or regeneration, potentially reducing postoperative complications. In addition, the observed inter‐individual variability may warrant further investigation into its potential relevance to personalized injury risk and underlying genetic influences.

This study has several limitations. First, porcine knees were used instead of human specimens. However, previous studies [10] have demonstrated substantial anatomical and biomechanical similarities between porcine and human ACLs, supporting the translational relevance of our findings. Nonetheless, future studies should aim to obtain subject‐specific human data to guide surgical planning and graft design more precisely. Second, the finite element model was developed based on a single specimen, and no statistical analysis was performed for this part of the study. However, the one subject‐based model allows strict control of confounding variables and negates any impact of subject differences on the studied characteristic. Therefore, statistical analysis was not considered necessary in this part.

## CONCLUSION

At the ACL insertion, the area proportion of the direct region was significantly greater than that of the indirect region, and the AM bundle occupied a larger proportion than the PL bundle. The tensile Young's modulus of the indirect region was significantly higher than that of the direct region, whereas no significant difference was observed between the AM and PL bundles. Regional variations in area proportion and material properties substantially affected the insertion's load magnitude and stress distribution. Incorporating region‐specific material heterogeneity notably increased the force borne by the indirect insertion. A more balanced area proportion between the direct and indirect regions led to a more uniform stress distribution. These findings highlight the importance of accurately replicating region‐specific material properties and area proportions in ACL reconstruction to restore appropriate mechanical function and reduce the risk of postoperative failure of the ligament‐bone interface.

## AUTHOR CONTRIBUTIONS

**Kaixin He**: Data curation, methodology, formal analysis, writing—original draft, writing—review and editing; **Qingqing Yang**: Data curation, methodology, formal analysis, writing—review and editing; **Qinyi Shi**: Data curation, writing—review and editing; **Huizhi Wang**: Conceptualization, methodology, formal analysis, funding acquisition, supervision, writing—review and editing; **Cheng‐Kung Cheng**: Conceptualization, funding acquisition, supervision, writing—review and editing.

## CONFLICT OF INTEREST STATEMENT

The authors declare no conflicts of interest.

## ETHICS STATEMENT

The Institutional Animal Care and Use Committee in Shanghai Jiao Tong University approved the project proposal (202101260).

## Data Availability

The data that support the findings of this study are available on request from the corresponding author.
